# Initiating change locally in bullying and aggression through the school environment (INCLUSIVE): study protocol for a cluster randomised controlled trial

**DOI:** 10.1186/1745-6215-15-381

**Published:** 2014-09-30

**Authors:** Chris Bonell, Elizabeth Allen, Deborah Christie, Diana Elbourne, Adam Fletcher, Richard Grieve, Rosa LeGood, Anne Mathiot, Stephen Scott, Meg Wiggins, Russell M Viner

**Affiliations:** Institute of Education, 20 Bedford Way, London, WC1H 0AL UK; London School of Hygiene & Tropical Medicine, Keppel Street, London, WC1E 7HT UK; UCL Hospitals NHS Foundation Trust, 250 Euston Rd, London, NW1 2PG UK; Cardiff School of Social Sciences, Cardiff University, Glamorgan Building, King Edward VII Avenue, Cardiff, CF10 3WT UK; UCL Institute of Child Health, 30 Guilford St, London, WC1N 1EH UK; Institute of Psychiatry, Kings College London, 16 De Crespigny Park, London, SE5 8AF UK

**Keywords:** Bullying, Cluster randomised trial, School intervention, Violence prevention, Adolescent

## Abstract

**Background:**

Systematic reviews suggest that interventions that address school organisation are effective in reducing victimisation and bullying. We successfully piloted a school environment intervention modified from international studies to incorporate ‘restorative justice’ approaches. This trial aims to establish the effectiveness and cost-effectiveness of the INCLUSIVE intervention in reducing aggression and bullying in English secondary schools.

**Methods:**

Design: cluster randomised trial.

Participants: 40 state-supported secondary schools. Outcomes assessed among the cohort of students in year 8 (n = approximately 6,000) in intervention year 1.

Intervention: INCLUSIVE is a school-led intervention which combines changes to the school environment with the promotion of social and emotional skills and restorative practices through: the formation of a school action group involving students and staff supported by an external facilitator to review local data on needs, determine priorities, and develop and implement an action plan for revising relevant school policies/rules and other actions to improve relationships at school and reduce aggression; staff training in restorative practices; and a new social and emotional skills curriculum. The intervention will be delivered by schools supported in the first two years by educational facilitators independent of the research team, with a third locally facilitated intervention year.

Comparator: normal practice.

Outcomes: primary: 2 primary outcomes at student level assessed at baseline and at 36 months:

1. Aggressive behaviours in school: Edinburgh Study of Youth Transitions and Crime school misbehaviour subscale (ESYTC)
2. Bullying and victimisation: Gatehouse Bullying Scale (GBS)

Secondary outcomes assessed at baseline, 24 and 36 months will include measures relating to the economic evaluation, psychosocial outcomes in students and staff and school-level truancy and exclusion rates.

Sample size: 20 schools per arm will provide 90% power to identify an effect size of 0.25 SD with a 5% significance level.

Randomisation: eligible consenting schools will be randomised stratified for single sex versus mixed sex schools, school-level deprivation and measures of school attainment.

**Discussion:**

The trial will be run by independent research and intervention teams and supervised by a Trial Steering Committee and a Data Monitoring Committee (DMC).

**Trial registration:**

Current Controlled Trials ISRCTN10751359 (Registered 11 March 2014)

## Background

The prevalence and harms of aggressive behaviours among youth make addressing them a public health priority [1–4]. The World Health Organisation considers bullying to be a major adolescent health problem, defining this to include the intentional use of physical or psychological force against others [5]. This includes verbal and relational aggression that aims to harm the victim or their social relations, such as through spreading rumours or purposely excluding them [6, 7]. The prevalence of bullying among British youth is above the European average [8], with approximately 25% of young people reporting that they have been subjected to serious peer bullying [9]. There are marked social gradients, with both family deprivation and school-level deprivation increasing the risk of experiencing bullying [10]. Bullying most commonly occurs in schools [11, 12] and prevalence varies significantly between schools [13–16].

Being a victim of peer bullying is associated with an increased risk of: physical health problems [17]; engaging in health risk behaviours such as substance use [18–20]; long-term emotional, behavioural and mental health problems [21–23]; self-harm and suicide [24]; and poorer educational attainment [25, 26]. Students who experience physical, verbal and relational bullying on a regular basis tend to experience the most adverse health outcomes [27]. There is also evidence suggesting that childhood exposure to bullying and aggression may also influence life-long health through biological mechanisms [28]. The perpetrators of peer bullying are also at greater risk of a range of adverse emotional and mental health outcomes, including depression and anxiety [8, 13].

Bullying is also often a precursor to more serious violent behaviours commonly reported by British youth. One UK study of 14,000 students found that 1 in 10 young people aged 11 to 12 reported carrying a weapon and 8% of this age group admitted they had attacked another with the intention to hurt them seriously [29]. By age 15 to 16, 24% of students report that they have carried a weapon and 19% reported attacking someone with the intention to hurt them seriously [29]. Inter-personal violence can cause physical injury and disability, and is also associated with long-term emotional and mental health problems. There are also links between aggression and anti-social behaviours in youth and violent crime in adulthood [30, 31]. There is increasing concern because low-level provocation and aggressive behaviours in secondary schools are educationally disruptive, emotionally harmful, reduce educational attainments and later life-chances, and can lead to more overt physical aggression over time [32–34]. The economic costs to society as a whole due to youth aggression, bullying and violence are extremely high. For example, the total cost of crime attributable to conduct problems in childhood has been estimated at about £60 billion a year in England and Wales [35].

### School-based interventions

Reducing aggression, bullying and violence in British schools has been a consistent priority within recent public health and education policies [36–38]. The 2009 Steer Review concluded that schools’ approaches to discipline, behaviour management and bullying prevention vary widely and are rarely evidence-based, and that further resources and research are urgently needed to combat aggressive behaviours and other conduct problems [34]. There is, therefore, a pressing need to determine which interventions are effective in addressing bullying and aggression in schools, and to scale up such interventions across local and national school networks.

A number of systematic reviews assess school-based interventions to address bullying and aggression. Interventions that promote change across school systems and addressed different levels of school organisation, that is ‘whole-school’ or ‘school environment’ interventions, are particularly effective in reducing victimisation and bullying than curriculum interventions [39–41]. The effectiveness of such interventions may be because they address bullying as a systemic problem meriting an ‘environmental solution’ [39]. Whole-school interventions are also inherently universal in reach and likely to provide a cost-effective and non-stigmatising approach to preventing bullying [40]. This is in keeping with other evidence from the UK and internationally which shows that schools promote health most effectively when they are not treated merely as sites for health education but also as physical and social environments which can actively support healthy behaviours and outcomes [42, 43].

These school environment interventions thus take a ‘socio-ecological’ [44] or ‘structural’ [45] approach to promoting health whereby behaviours are understood to be influenced not only by characteristics of individuals, but also the wider social context. A recent National Institute of Health Research (NIHR)-funded systematic review of the health effects of the school environment found evidence from observational and experimental studies that modifying the way in which schools manage their ‘core business’ (teaching, pastoral care and discipline) can promote student health and potentially reduce health inequalities across a range of outcomes, including reductions in violence and other aggressive behaviours [43]. Other outcomes that are improved by school environment interventions include mental health and physical activity and reduced substance use including alcohol, tobacco and drugs [43].

School environment interventions that impact on a range of health risk behaviours including aggression are likely to be one of the most efficient ways of addressing multiple health harms in adolescence, due to their potential for modifying population-level risk as well as their reach and sustainability [43]. Multiple risk behaviours in adolescence are subject to socio-economic stratification, and are strongly associated with poor health outcomes, social exclusion, educational failure and poor mental health in adult life [46]. A recent King’s Fund report on *The Clustering of Unhealthy Behaviours Over Time*, emphasised the association of multiple risk behaviours with mortality and health across the life-course, and the policy importance of reducing multiple risk behaviours among young people through new interventions that address their common determinants [47].

The INCLUSIVE intervention under trial here has been particularly informed by two international evidence-based school environment programmes. First, the Aban Aya Youth Project (AAYP) is a multi-component intervention, enabling schools to modify their social environment as well as delivering a social skills curriculum. This approach was designed to increase social inclusion by ‘rebuilding the village’ within schools serving disadvantaged, African-American communities. To promote whole-school institutional change at each school, teacher training was provided and an action group was established (comprising both staff and students) to review policies and prioritise actions needed to foster a more inclusive school climate. For boys, the intervention was associated with significant reductions in the growth in violence and aggressive behaviour [48]. The intervention also brought benefits in terms of reduced sexual risk behaviours and drug use, as well as provoking behaviour and school delinquency. Second, the Gatehouse Project in Australia also aimed to reduce health problems via changing the school climate and promoting security, positive regard and communication among students and school staff. As with the AAYP, an action group was convened in each school, facilitated by an external ‘critical friend’ and informed by data from a student survey, alongside a social and emotional skills curriculum. A cluster randomised controlled trial (RCT) found consistent reductions in a composite measure of health risk behaviours, which included violence and anti-social behaviour [49, 50].

INCLUSIVE extends the AAYP and Gatehouse interventions by including ‘restorative justice’ approaches. The Steer Review in 2009 called for English schools to consider adopting more restorative approaches to prevent bullying and other aggressive behaviour to help minimise the harms associated with such problems [34]. The central tenet of such approaches is to repair the harms caused to relationships and communities rather than merely assign blame and enact punishment. Such approaches have now been adapted for use in schools and can operate at a whole-school level, informing changes to disciplinary policies, behaviour management practices, and how staff communicate with students in order to improve relationships, reduce conflict and repair harm. An example of such restorative practice currently employed in schools is the use of ‘circle time’ to develop and maintain good communication and relationships [51]. Restorative ‘conferencing’ can also be used in schools to deal with more serious incidents [51].

Restorative approaches have only been evaluated using non-random designs, although such studies do suggest that the restorative approach is a promising one in the UK [52–54] and internationally, particularly when implemented at the whole-school level [55–57]. For example, in England and Wales, the Youth Justice Board evaluated the use of restorative approaches at twenty secondary schools and six primary schools, and reported significant improvements regarding students’ attitudes to bullying, and reduced offending, and victimisation in schools that adopted a whole-school approach to restorative practice. Restorative approaches thus appear to have the potential to complement school-environment interventions such as Aban Aya and the Gatehouse Project. They offer a highly promising way forward for reducing aggressive behaviours among British youth. A recent Cochrane review found no RCTs of interventions employing restorative approaches to reduce bullying in schools and recommended that this should be a priority for future research [58]. If trialled and found to be effective, such a universal school-based approach could be scaled up to reach very large numbers of young people and deliver significant population-level health improvements.

### Findings from the INCLUSIVE pilot study

The evidence above demonstrates that bullying and aggression are highly prevalent in English schools, and generate health harms and inequalities, educational and other harms, and economic costs. While existing systematic reviews suggest ‘whole-school’ interventions are an effective approach to addressing these problems, the recent Cochrane review [58] recommends further trials in this area examining restorative practices. The INCLUSIVE intervention addresses these points and has been successfully piloted, funded for 20 months (July 2011 to February 2013) through a commissioned funding call from the UK NIHR Health Technology Assessment (HTA). Criteria were agreed for progression to a full trial, with further funding for a phase III trial of a three-year intervention being dependent on a new funding application. Intervention funding was provided by the Paul Hamlyn Foundation, the Big Lottery Fund, and the Coutts Charitable Trust.

We undertook a cluster RCT in eight mixed-sex secondary schools in London and south-east England, purposively sampled to ensure diversity with regard to Ofsted rating and rate of eligibility for free school meals (four intervention, four comparison) with integral process evaluation. The aim was to assess the feasibility and acceptability of the INCLUSIVE intervention and trial methods over one academic year (whereas INCLUSIVE was designed as a three-year intervention). The objectives of the study were to: (1) examine the feasibility and acceptability of delivering and trialling the intervention according to pre-specified criteria agreed with the HTA; (2) explore participants’ experiences of implementing and trialling the intervention and how this varied according to school context to refine the intervention and trial methods; and (3) pilot indicative primary outcomes (aggressive behaviour measures), other outcomes and economic evaluation methods.

All pre-specified feasibility and acceptability criteria were met (objective 1) and the process data indicated that all intervention components, the trial design and methods were feasible and acceptable (objective 2). Qualitative data suggested that student participation may be a core component in improving relationships and engagement across the school. Appropriate outcome measures and economic methods were identified (objective 3): the Gatehouse Bullying Scale (GBS) and the Edinburgh Study of Youth Transitions and Crime (ESTYC) school misbehaviour subscale were acceptable, discriminating and reliable measures of bullying and aggression in this context. Pilot economic analyses support the use of the Child Health Utility 9D (CHU9D) scale with this population and the feasibility of cost-utility analysis. Analysis of outcomes in the pilot showed that confidence intervals encompassed potential intervention benefits. There was no evidence of harm.

We were then successful in obtaining further NIHR funding from the Public Health Research programme (PHR) to undertake a large-scale cluster RCT to examine the effectiveness and cost-effectiveness of the INCLUSIVE intervention. Intervention funding was obtained from the Educational Endowment Fund (EEF), which also funded an independent evaluation of effects on educational attainment to be conducted by the University of Manchester.

### Research questions

RQ1. Is the INCLUSIVE intervention implemented over three school years more effective and cost-effective than standard practice in reducing bullying and aggression among 12- to 15-year olds in English secondary schools?

RQ2. Is the INCLUSIVE intervention more effective than standard practice in improving students’ quality of life (QoL), well-being, psychological function and attainments, and reducing school exclusion and truancy, substance use, sexual risk, National Health Service (NHS) use, police contacts among students, and improving staff QoL and attendance and reducing burn-out?

RQ3. What pre-hypothesised factors moderate and mediate the effectiveness of the INCLUSIVE intervention; including, do effects vary by socio-economic status and sex?

## Methods

The trial is a 3-year cluster randomised controlled trial with integral economic evaluation and process evaluation in 40 schools across south-east England, with schools as the unit of allocation.

### Study population

INCLUSIVE is a universal intervention, aimed at all 11- to 16-year olds in participating secondary schools in England. While the intervention will have effects on the whole school, our study population of students will be those at the end of year 7 (age 11 to 12 years) at baseline and at the end of year 10 at 36-month follow-up (age 14 to 15), as well as all school teaching and teaching assistant staff. All students in the school in that year and all teaching staff will be surveyed at each time-point, not only those who participated at baseline.

### Inclusion/Exclusion criteria

Eligible schools are those:

(i). Secondary schools within the state education system (including community, academy or free schools, and mixed or single sex) in south-east England. We will take the widest definition of a ‘state school’ and will only exclude private schools, schools exclusively for those with learning disabilities and pupil referral units. The latter two will be excluded as it is unlikely that INCLUSIVE will be appropriate for their populations.
(ii). Ofsted rating (most recent) of ‘requires improvement’/‘satisfactory’ or better; we will exclude schools with an ‘inadequate’/‘poor’ Ofsted rating because such schools are subject to special measures which are likely to impede INCLUSIVE delivery.

Note there are no inclusion/exclusion criteria for students.

### Recruitment

Schools will be recruited from secondary schools in Greater London and the surrounding counties (Surrey, Kent, Essex, Hertfordshire, Buckinghamshire, and Berkshire) with a maximum travel time of one hour from the study centres in London. To aid recruitment, we will partner with existing schools networks such as the UCL Partners Schools Network, the Institute of Education Teaching Schools and schools that are part of our collaborating schools network, Challenge Partners. We will approach approximately 500 eligible schools, initially by letter and Email with a telephone follow-up, complying with good practice and research governance for undertaking studies within the education system.

### Randomisation

Eligible schools whose head-teacher gives informed written consent to participate will be allocated with a 1:1 ratio between intervention and control arms. Stratified randomisation will be undertaken remotely by the Clinical Trials Unit (CTU) at the London School of Hygiene & Tropical Medicine (LSHTM). To promote baseline equivalence, we will stratify by key school-level determinants of violence:

- Single sex versus mixed sex school.
- School-level deprivation, as measured by percentage of students eligible for free school meals (low/moderate 0 to 23%; high >23%, with 23% being the median for England).
- School ‘best eight value added’ in GCSE exams (above and below median for England of 1,000). Value added (VA) score is a school-level measure of students’ attainment in public exams adjusting for their attainment on entry to the school. We use VA rather than Ofsted ratings for schools as there is better evidence for VA being associated with violence rates [59].

Schools will be allocated randomly within each of these eight strata.

Protecting against selection bias:

1. School level: the randomisation schedule will be drawn up once the schools have consented and after the baseline survey, thus guarding against selection biases at entry of clusters to the trial. The randomisation may occur sequentially in groups of 10 schools, should there be any delays with baseline surveys in some schools. As with most social intervention trials, schools, their students, teachers and other staff cannot be ‘blinded’ to allocation status. However, fieldwork staff will be blinded to allocation as will data-input staff. Analysis of follow-up quantitative data will be undertaken blind to allocation.

Retention of control schools will be maximised by ensuring regular senior liaison and provision of participation incentives (£500 per school).

**Figure 1:**
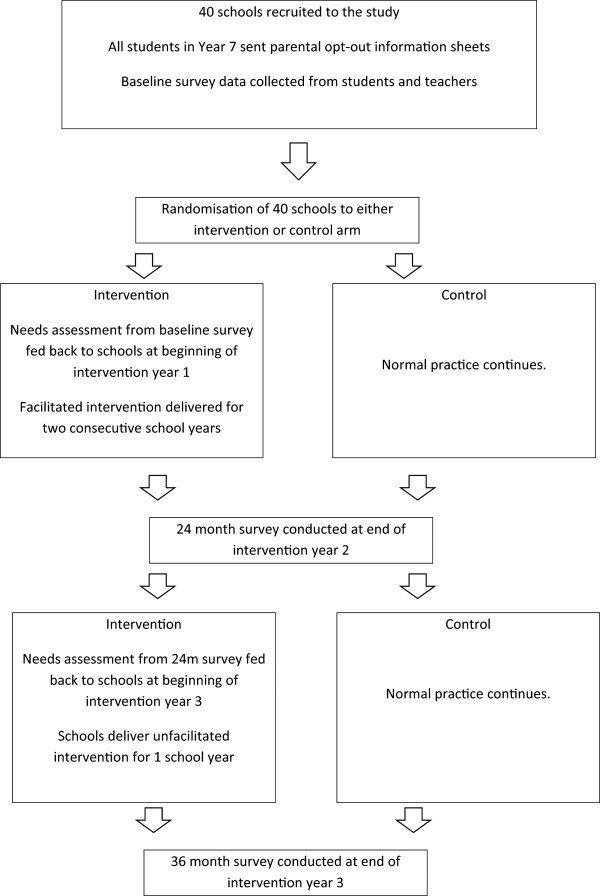
**Flow chart showing intervention and control treatment.**

(2) Student level: we had very high student participation in our pilot study: 96% of eligible at baseline and 93% at follow-up. To minimise bias, we will use in-school, mail and telephone contacts to try to include all enrolled students absent at either baseline or follow-up questionnaires. Note we will not attempt to follow-up students who have left the school.
  A flow chart of recruitment and intervention and control treatment is shown in Figure 1.

Intervention and comparison groups:

1. Intervention:

The INCLUSIVE intervention is intended principally to augment rather than to replace existing activities (for example, training, curricula, and so on) in intervention schools. However, it is intended to replace existing non-restorative disciplinary school policies and practices where restorative approaches are deemed by the action group to be more appropriate.

The facilitated phase provides the following inputs:

i). Annual surveys of local needs and assets (including bullying, aggression, prevalence and determinants) and progress in addressing these.
ii). Support from an external expert education facilitator trained in facilitating INCLUSIVE.
iii). Social and emotional learning curriculum resources.
iv). Staff training in restorative practices provided by the education facilitators and comprising a short introduction and subsequent half day for all staff (focused on introducing them to restorative practices, such as ‘circle-time’, to promote positive relationships and communication, plus enhanced three-day training course in restorative practices targeting five to ten staff at each school, including training in formal ‘conferencing’ to deal with more serious incidents via bringing together students, parents and/or staff.)

These inputs will enable schools during all three years to convene an action group, which comprises (at a minimum):

1. Six students
2. Six staff, including at least one Senior Management Team (SMT) member and one member of each of the teaching, pastoral and support staff

Membership from specialist health staff, such as the school nurse and/or local child and adolescent mental health services staff, are desirable but optional. The action group must meet at least six times per school year (that is approximately once every half-term).

The action group develops an action plan that coordinates delivery of the following intervention outputs:

i). Reviewing and revising *school rules and policies* relating to discipline, behaviour management and staff-student communication.
ii). Implementing *restorative practices* throughout the school. Restorative practices include ‘circle-time’ (which brings students together with their teacher during registration periods or other lessons to maintain good relationships, or be used to deal with specific problems) and ‘conferencing’ (used to deal with more serious incidents and brings together relevant staff, students, parents and, where necessary, external agencies).
iii). Additional *tailored actions* to address local priorities.
iv). Delivering the *social and emotional skills curriculum* for years eight to ten. The curriculum targets students in years eight to ten who receive five to ten hours teaching and learning per year on restorative practices, relationships, and social and emotional skills based on the Gatehouse Project curriculum. The curriculum is designed as a set of learning modules which schools can address using our own or existing materials if these aligned with our curriculum. Modules cover: establishing respectful relationships in the classroom and the wider school; managing emotions; understanding and building trusting relationships; exploring others’ needs and avoiding conflict; and maintaining and repairing relationships. Informed by the needs-assessment data, schools will tailor the curriculum to their needs and could deliver modules either as ‘stand-alone’ lessons, for example within Personal, Social and Health Education (PSHE), and/or integrated into various subject lessons (for example, English).

The intervention enables local tailoring, informed by the needs survey and other local data sources. These locally adaptable actions occurred within a standardised overall process with various core standardised intervention elements, such as the staff training in restorative practices; review and revision of school rules and policies; and the social and emotional skills curriculum. This balance of standardisation and flexibility is a common practice in complex interventions, enabling a balance between fidelity of the core components with local adaption [60]. This allows schools to build on their current good practice, and also encourages students and staff to develop ownership of the work, which may be a key factor in intervention effects. To support this, the facilitator works with schools to ensure all members of the action group are supported to identify and undertake locally determined actions to improve the school environment.

Internally facilitated intervention year: the third intervention year will be identical to the externally facilitated intervention described above, with the exception that there will be no provision of external facilitation. One of the roles of the external facilitator over the two facilitated years will be to ensure the school action group and SMT develop the capacity to undertake this internal facilitation in the third year.

2. Comparator - control schools:

Schools randomised to the control group will continue with normal practice for the school in question and receive no additional input. They will be provided with £500 (to cover administrative costs and/or provide cover for staff involvement in organising data collection) and at the end of the study be offered a brief report of the survey data collected at the school. Control schools are free to engage in actions to reduce bullying and aggression but the contract signed with head-teachers will preclude their engaging in a facilitated whole-school programme similar to INCLUSIVE during the period of the trial. We will examine control schools’ policies and practices related to bullying and aggression.

### Endpoints of the study

#### Primary outcomes

The primary outcome will be an assessment of experience of violence and bullying measured using 2 scales at 36 months through student survey self-reports. As is conventional in trials of interventions addressing violence and aggression in schools, we will rely on self-reports from students, rather than observations or teacher reports, because of the impracticality and greater likelihood of bias respectively of the latter two. The primary outcomes measures include one measure of bullying victimisation and one measure of perpetration of aggressive behaviours that were shown to be reliable and valid in our pilot study:

- *Gatehouse Bullying Scale* (*GBS*). The GBS [49] is a short, reliable tool to measure the occurrence of bullying victimisation in schools. This measure was designed by one of our collaborators (LB) and has been shown to be related to other measures of social attachments, school engagement, and anxiety and depressive symptoms. The scale has 12 items, and asks about being the subject of recent teasing, name-calling, rumours, being left out of things and physical threats or actual violence from other students in the last 3 months. Each section asks about the recent experience of that type of bullying (‘yes’ or ‘no’), how often it occurred, and how upset the student was by each type of bullying [49, 61].
- *Edinburgh Study of Youth Transitions and Crime* (*ESYTC*) *school misbehaviour subscale*. The ESYTC measures several domains of violence and aggression at school [62].

#### Secondary outcomes

These will include our aggression/bullying measures (GBS and ESYTC) measured at 24 months and other outcomes measured at both 24 and 36 months:

- Student-self-report outcomes: these will be measured through student survey self-reports:
- Paediatric quality of life inventory (PedsQL) version 4.0 will be used to assess overall QoL. The 30-item PedsQL [63] has been shown to be a reliable and valid measure of QoL in normative adolescent populations. It consists of 30 items representing five functional domains: physical, emotional, social, school and well-being, and yields a total QoL score, two summary scores for ‘Physical Health’ and ‘Psychosocial Health’ and three subscale scores for ‘Emotional’, ‘Social’, and ‘School’ functioning.
- Psychological function and well-being;
- The Strengths and Difficulties Questionnaire (SDQ) [61] is a brief screening instrument for detecting behavioural, emotional and peer problems and pro-social strengths in children and adolescents. It is brief, quick to complete, and validated in national UK samples.
- Short Warwick-Edinburgh Mental Well-Being Scale (SWEMWBS) [64] is a seven-item scale designed to capture a broad concept of positive emotional well-being including psychological functioning, cognitive-evaluative dimensions and affective-emotional aspects, with a total ‘Well-Being Index’ generated.
- Risk behaviours;
- Substance use. Validated age-appropriate questions taken from national surveys and/or previous trials will be used to assess smoking (smoking in previous week; ever smoked regularly), alcohol use (use in previous week; number of times really drunk; binge drinking) and illicit drug use (last month; lifetime use).
- Sexual risk behaviours: age of sexual debut and use of contraception at first sex may be examined by measures used in the Ripple trial [65]. We will consult with schools about the acceptability of asking these questions at follow-up (year ten).
- Use of NHS services: self-report use of primary care, accident and emergency, other service in past 12 months.
- Contact with police will be assessed using the Young People’s Development Programme (YPDP) evaluation measure [66], which asks whether the young person has been stopped, told off, or picked up by the police in the last 12 months.

(ii) Student-level data collected from schools:

1. School attendance will be measured via routine school data on each student expressed as number of half days absent; for which we will seek students’ informed consent to access.
2. Educational attainment: this will be assessed by an independent team based at the University of Manchester drawing on routine data.

(iii) Individual staff-level outcomes. We will measure the following secondary outcomes through survey self-reports from teachers and teaching assistants:

1. Staff attendance will be measured via routine school data on each staff-member expressed as number of half days absent; for which we will seek staff-members’ informed consent to access.
2. Staff QoL will be measured using the Short Form (SF)-12 version 2 Health Survey [67], a brief well-validated measure of adult health-related QoL.
3. Staff stress and burnout will be measured using the Maslach Burnout Inventory [68], an established scale which uses a three-dimensional description of exhaustion, cynicism, and inefficacy.

(iv) School-level outcomes: routinely-collected data on school rates of temporary and permanent exclusions.

Student surveys will be conducted in exam conditions in schools, maximizing privacy. All students in the school in that year and all teaching and teaching assistant staff will be surveyed at each time-point, not only those who participated at baseline. Paper-based questionnaires will be completed confidentially in a 45-minute class session devoted to the purpose. Field workers will supervise the class completing the questionnaire, with the teacher present (for disciplinary purposes) but unable to see the questionnaires. The field-workers will assist students with questions that they do not understand and ensure students complete as much of the questionnaire as possible. Note that students with mild learning difficulties or with limited command of written English will be supported to complete the questionnaires by fieldworkers.

We will ask students in intervention schools involved in qualitative interviews whether their reporting (as opposed to their experience) of bullying and aggression might have been affected by the intervention.

### Power and sample size

The average English school has approximately 190 students per year, although this varies across schools. A systematic review of school-based secondary preventive interventions to prevent violence [69] reported a pooled effect size of 0.41 on measures of aggressive behaviour. Effect sizes for aggressive behaviour from similar interventions approximate 0.3 to 0.4 SDs in males. Recent data from three large UK school cohorts [70] suggest that intra-cluster correlation coefficients (ICC) for aggression and bullying outcomes vary between 0.01 and 0.03

We propose to recruit sufficient participants to detect a difference between groups of 0.25 SD with 90% power and a 5% level of significance. This is considered to represent a moderate size of effect and in line with the effect sizes seen in the literature.

Conservatively, taking an ICC of 0.04 and 150 students per school, a trial involving 20 schools per arm will provide 90% power to identify an effect size of 0.25 SD with a 5% significance level. If two schools per arm (that is 10%) were to be lost to follow-up over the course of the trial, we would still have 80% power to detect an effect size of 0.25 SD.

The total student sample size will be approximately 6,000. As we will be surveying all young people in the relevant school year at each follow-up, this sample is likely to remain similar across the study.

### Economic evaluation

The aim of the economic evaluation is to assess the costs, consequences and cost-effectiveness of the INCLUSIVE intervention compared with standard school-based practices for managing aggression.

The primary economic evaluation will take the form of a within-trial cost-consequence analysis, with a secondary analysis that will report relative cost-utility with health outcomes expressed in terms of Quality-Adjusted Life-Years (QALYs), as recommended by the National Institute for Health and Care Excellence (NICE)’s public health methods guidance.

This NICE guidance also recommends that the base-case cost-effectiveness estimate is presented from a public sector perspective as this allows the costs and benefits of more than one central/local government body to be taken into account. This statement is particularly pertinent to INCLUSIVE as the costs of implementing it are likely to fall on the educational sector, yet there are potential cost implications for sectors such as the NHS, the police and the judiciary through reduced anti-social behaviour.

The costs to the education sector include cost of the facilitator to deliver the intervention and the cost of staff time. The facilitator costs for the delivery of the intervention will be collected using log sheets. The impact on staff time for training and delivering bullying policy will be obtained as part of the process evaluation. It is possible that the intervention might offset some of the staff time related to dealing with pupil aggression or bullying behaviour and this will be captured as part of the teacher survey. It might also impact on teacher health and we will capture this by valuing the number of days off work, which will be captured as a secondary outcome measure. The implications for NHS resource use and policing will be identified with specific questions in the student survey and valued accordingly. The time horizon will capture costs and outcomes within the trial.

Changes in health-related QoL (as expressed using QALYs) will be measured from the study participants’ (that is student’s/teacher’s) perspective.

The *Child Health Utility* (*CHU*) *9D measure* (CHU-9D) [71] will be used to assess student’s health-related QoL as part of the economic evaluation. The CHU-9D is a validated age-appropriate measure that was explicitly developed using children’s input and has been suggested to be more appropriate and function better than other health utility measures for children and adolescents. For teachers, we will use the SF-12 for this purpose [67]. Student and teacher utility values will be collected (at baseline and at follow-up surveys at 24 and 36 months) using the CHU-9D and by converting the SF-12 questionnaires respectively.

### Process evaluation

Data will be used to examine intervention implementation and receipt and examine possible causal pathways in order to facilitate interpretation of outcome data. In line with Medical Research Council (MRC) guidance on complex interventions, this component of the trial will also enable refinement of the intervention logic model. Informed by existing frameworks, the process evaluation will examine the following:

#### Trial context

We will assess the context within the intervention and control arms, including what other relevant services and practices operate, such as the nature of school discipline systems, staff training, social skills curricula and student participation in decision-making. This will draw on annual: interviews with intervention facilitators (n = 5); telephone interviews with action-team members (n = 2 per school) in intervention schools; interviews with the Senior Leadership Team (SLT) (n = 1 per school) and other staff (n = 2 per school) in intervention and control schools; and 2 focus group discussions (FGDs) with students and one FGD with staff in 8 randomly selected intervention and control schools (purposively sampled by students participation, gender and age and staff participation and role), which will also allow us to explore mechanisms of actions.

#### Trial arm fidelity

We will assess the fidelity with which INCLUSIVE is delivered in each school. In addition to the above sources, we will draw on: annual structured quantitative researcher observational data of a random selection from each school of one action team meeting (n = 20), staff training (n = 20) and one curriculum session (n = 20); structured diaries of action team meetings and staff training maintained by intervention facilitators in each school; qualitative data from action-team minutes (from 10 randomly selected schools in the full trial). We will assess fidelity and acceptability rates for each facilitator.

#### Participation, reach and dose

We will assess the extent to which students and staff are involved in or in receipt of intervention processes and outputs. This will draw on quantitative data from 24- and 36-month follow-up surveys of students, staff and action group members. The last of these will also assess the extent to which members felt empowered to participate in decision-making using a modified version of the Learner Empowerment Scale [72].

#### Reception and responsiveness

We will assess the *experiences* of participation in INCLUSIVE and in school environments shaped by this, to assess *acceptability and any barriers or facilitators to this*. This will draw on the annual interviews with action-team members (n = 2 per school) in intervention schools; interviews with SLT (n = 2 per school) and other staff (n = 2 per school) in intervention and control schools; and FGDs with students in 8 randomly selected intervention schools described above.

#### Intermediate outcomes

To assess possible intervention causal pathways and examine whether these mediate intervention effects in order to assess and refine our logic model, we will use two measures that examine students’ perception of the school environment and their connection to the school:

- Beyond Blue School Climate Questionnaire (BBSCQ) [73] which will be used to measure students’ perceptions of the school climate. It consists of twenty-eight items which produce an overall score and also assesses four key domains of school climate (subscale): supportive teacher relationships, sense of belonging, participative school environment, and student commitment to academic values.
- Student reports of anti-school actions will be assessed using the ESYTC Self-Reported Delinquency (SRD) subscale. Involvement with anti-school peer groups will be assessed using a single item measure previously used in the YPDP evaluation measure.

### Analyses

#### Outcome analyses

All primary analyses will be carried out according to the principle of intention-to-treat (ITT) and using multilevel modelling to take into account clustering at the school level. The primary analysis will be a repeat cross-sectional analysis that includes data from all students at both time points for two main reasons: (1) the intervention is a whole school intervention and, based on a school-level theory of change, is expected to impact on all pupils, not just on those pupils who were present at baseline; (2) the literature suggests that in cluster randomised trials, when migration into or out of the clusters is high over time, the baseline cohort may not remain representative of the cluster and therefore repeated cross-sectional analysis is preferred to minimise bias. Based on our pilot data and existing research on student mobility, we anticipate student turn-over of up to 25% in some schools over 36 months. Because of this we will use multilevel analyses that include all students at all time-points, which essentially provides a repeat cross-sectional analysis with a nested longitudinal cohort.

Data will be analysed by appropriate multivariate regression models, fitting pre-hypothesised potential confounders as covariates. Note that data on ethnicity and socio-economic status will be collected by self-report from students. Both primary outcomes will be fully analysed and reported separately, using separate multi-level models. A small number of secondary analyses based on explicit hypotheses, for example, subgroup effects/causal pathway analyses will be specified in advance. These secondary analyses will include a longitudinal analysis of pupils present at both baseline and follow-up, with further analyses using individual-level baseline data to explore the implications of missing individual-level outcome data.

Secondary analyses will include staff outcomes and will be carried out according to the principle of ITT using the same approach to modelling as described for the student outcomes. Secondary analyses will also examine moderators and mediators. We will examine whether intervention effects are moderated by individual-level gender and socio-economic status measured using the Health Behaviours in School-aged Children (HBSC) Family Affluence Scale [74] and sex, as well as by school-level stratifying factors (single sex versus mixed sex school; school-level deprivation; value added strata); and facilitator, though these analyses may be underpowered. We will examine whether intervention effects are mediated by process and intermediate outcome measures. Other such analyses will be informed by hypotheses derived from analysis of qualitative data.

#### Economic analyses

The primary economic evaluation will be a cost-consequence analysis. We will undertake a cost-utility analysis as a secondary analysis. These analyses will be linked and use of both is consistent with NICE methods guidance for evaluating public health interventions. We propose using a multi-level modelling approach with random intercepts to estimate the mean and standard errors for both cost and effects along with the covariance matrix. From these data mean incremental net benefit and confidence intervals will then be estimated. Missing data will be handled using multiple imputation.

#### Process evaluation analyses

Qualitative data will be entered into the data analysis package NVivo (QSR International (UK) Limited, Vanguard House, Keckwick Lane, Daresbury, Cheshire, WA4 4AB, United Kingdom, Telephone: +44 (0) 1925 357 960) which will be used to manage and code data. Qualitative data from the process evaluation will be subjected to a thematic content analysis. Codes will be applied to transcripts, which identify key themes and how these inter-relate in order to develop an analytical framework. Each transcript will be coded to indicate the type of participant, school and date, allowing analytical themes to be explored in relation to different groups’ experiences and to compare processes across schools. Drawing on methods associated with ‘grounded theory’, we will make constant comparisons and examine deviant cases to refine our analysis. Analysis will explore implementation and receipt and contextual factors affecting these, as well as potential causal pathways in order to develop hypotheses to examine in secondary moderator and mediator analyses. Additionally, quantitative data from surveys and observations will be used in analyses of intervention fidelity and reach using simple descriptive statistics.

### Ethical issues

The study has been approved by the Institute of Education Research Ethics Committee (18/11/13 ref. FCL 566) and the University College London Research Ethics Committee (30/1/14, Project ID: 5248/001).

### Consent

Written consent will be obtained at school level (head-teacher) for random allocation and for intervention, and at the individual student, staff and intervention facilitator level for data collection. For students, written age-appropriate information sheets will be provided in class one to two weeks before the baseline survey, together with oral explanation by teachers. Written consent will be required from all participating young people, which will be collected immediately before conducting the baseline survey. Young people will also be asked to take home written information sheets for parents. Parents who do not wish their child to participate will be asked to notify this opt-out in writing using a prepared form.

### Confidentiality

All information collected during the trial will be kept confidential and adhere to the 1998 Data Protection Act.

### Risk, burdens and benefits

#### Benefits

If successful, the INCLUSIVE intervention will result in the following benefits:

1. Reduction of bullying and aggression which will be of benefit to all participants, the whole school, local communities and society in general.
2. Reduction in other health-risk outcomes (for example, substance use) and improvements in mental health, emotional well-being and QoL.
3. Reduction in costs to society related to bullying and aggression. These include reductions in NHS costs (related to violence and mental health problems), and in social costs including costs within the justice system.
4. Benefits to school staff through increased access to restorative training and an improved school environment, which may improve staff well-being and QoL.
5. Benefits to students who participate in the intervention, through opportunities for learning and improved self-efficacy.

#### Risks

There are no anticipated risks to participants or to schools. However, as in all interventions, there may be unanticipated risks. Harms will be assessed through examination of outcomes at 24 and 36 months. An independent Data Monitoring Committee (DMC) will examine any potential harms at 24 months. If any major harms are detected, the DMC will inform the Trial Steering Committee (TSC) who will decide what action should be taken.

It is possible that our approach may be ineffective, and its introduction in trial schools may prevent the use of more effective techniques to reduce aggression. Although some educational interventions to raise awareness of risk behaviours during adolescence have been shown to increase participation in these behaviours, we believe this is extremely unlikely in the case of this study because as our approach is based upon what is shown to be effective in systematic reviews. Because of the above, we believe that risks are minimal and that benefits justify the risks.

### Study governance

#### Trial documentation

Relevant trial documentation will kept for a minimum of 15 years.

#### Trial registration and conduct

The trial is registered with http://www.controlled-trials.com (ISRCTN 10751359); note that the ISRCTN for the pilot study was 88527078. As the trial is not within clinical settings nor using clinical samples nor using a medicinal product, there is no requirement to comply with the ‘The Medicines for Human Use (Clinical Trials) Regulations 2004’. We will follow the UK MRC Guidelines on Good Clinical Practice in Clinical Trials. Note that the chief Investigators (CI) and the majority of the other investigators have been trained in Good Clinical Practice for clinical trials.

#### Sponsor

The UCL Institute of Child Health, the employer of one of the CIs, will act as the sponsor of this trial.

TSC: the trial will be overseen by a TSC, including an independent chair (Professor Laurence Moore, University of Glasgow), at least two other independent members, Patient and Public Involvement representatives including young people and teachers involved in our pilot study, and an investigator representative of each institution involved in the research. Observers from the PHR programme will be invited to all TSC meetings. The TSC will meet six-monthly throughout the trial.

#### *Data Monitoring Committee*(*DMC*)

A DMC will be established independent of the investigators and of the TSC, but reporting to the TSC and (via the TSC) to the sponsors and the HTA programme. This will consist of an independent chair, a senior statistician and at least one other senior academic independent of the investigators. This will meet approximately yearly during the study. The DMC will monitor data for quality and completeness. Data quality, follow-up and trial monitoring will be facilitated through the development of a trial-specific database, including validation, verification, monitoring and compliance reports and follow-up report functionalities. The DMC will examine the results of an interim analysis at 24 months to consider any potential harms.

#### Study management

Russell Viner (RV) will direct the study together with Chris Bonell (CB) as co-CI. The intervention and research teams will be functionally independent. The research team will be managed by RV, CB and Anne Mathiot (AM), the trial manager. CB will direct the process evaluation.

The trial manager will have day-to-day responsibility for the conduct of the trial and the operations of the research team. The trial manager will report to the CIs and to a trial management group made up of RV, CB, AM together with the lead study statistician Elizabeth Allen (EA) and the lead for the intervention team, Meg Wiggins (MW). The trial management group will meet monthly throughout the study, and report to the Scientific Steering Committee (SSC) made up of all named investigators. The SSC will meet four- to six-monthly throughout the trial. Responsibility for data integrity and analysis will be held by the Clinical Trials Unit (CTU) at the London School of Hygiene & Tropical Medicine (LSHTM) (Diana Elbourne and EA). Responsibility for economic evaluation will be held by Richard Grieve at the LSHTM.

The intervention team will be managed by MW at the Institute of Education, together with Miranda Perry (MP), the intervention educational consultant who will direct day-to-day operation of the intervention and coordinate the educational facilitators.

## Discussion

The INCLUSIVE trial is part of a growing number of cluster randomised trials related to health but conducted within the education system in the UK. We have built upon evidence from US and Australian studies, modified the intervention to include restorative justice elements and shown feasibility and acceptability in a pilot study. This full trial of the INCLUSIVE intervention is a pragmatic ‘realist’ trial, evaluating not only the facilitated intervention (for the primary outcome) but also a further year of the intervention when continued by schools without external facilitation.

A number of elements of the trial will aid generalisability and scalability if shown to be effective. We have included a very wide range of participating schools, including all but schools whose current functioning we judge to be too low to be able to implement or benefit from the intervention. The intervention is flexible and can be tailored to each school’s needs and we have partnered with a number of school networks to facilitate future scalability.

Funding was obtained from both the health sector (through the National Institute of Health Research) and the education sector (the Education Endowment Fund). Each is funding separate teams to undertake the research (health sector) and the intervention (education sector).

The trial will be overseen by an independent TSC and DMC appointed by the main funders (NIHR)

## Trial status

At time of submission (2 June 2014) the trial has recruited all schools and is currently recruiting and surveying students for the baseline survey. Schools will be randomised after all baseline data are collected.

## Abbreviations

AAYP: Aban Aya Youth Project
BBSCQ: Beyond Blue School Climate Questionnaire
CHU: Child Health Utility
CHU-9D: Child Health Utility 9D
CI: chief investigators
CTU: Clinical Trials Unit
DMC: Data Monitoring Committee
EEF: Educational Endowment Fund
ESTYC: Edinburgh Study of Youth Transitions and Crime
FGD: focus group discussion
GBS: Gatehouse Bullying Scale
HBSC: Health Behaviours in School-aged Children
HTA: Health Technology Assessment
ICC: intra-correlation coefficients
ITT: intention-to-treat
LSHTM: London School of Hygiene & Tropical Medicine
MRC: Medical Research Council
NHS: National Health Service
NICE: National Institute for Health and Care Excellence
NIHR: National Institute of Health Research
PedsQL: Paediatric Quality of Life inventory
PHR: NIHR Public Health Research programme
PSHE: Personal, Social and Health Education
QALYs: Quality-Adjusted Life-Years
QoL: quality of life
RCT: randomised controlled trial
SDQ: Strengths and Difficulties Questionnaire
SF: Short Form
SLT: Senior Leadership Team
SMT: Senior Management Team
SRD: Self-Reported Delinquency
SWEMWBS: Short Warwick-Edinburgh Mental Well-Being Scale
TSC: Trial Steering Committee
VA: value added
YPDP: Young People’s Development Programme
